# A review on AI Safety in highly automated driving

**DOI:** 10.3389/frai.2022.952773

**Published:** 2022-10-03

**Authors:** Moritz Wäschle, Florian Thaler, Axel Berres, Florian Pölzlbauer, Albert Albers

**Affiliations:** ^1^IPEK—Institute of Product Engineering, ASE—Advanced Systems Engineering, Karlsruhe Institute of Technology (KIT), Karlsruhe, Germany; ^2^Virtual Vehicle Research GmbH, Graz, Austria; ^3^German Aerospace Center, Cologne, Germany

**Keywords:** AI Safety, systematic literature review, highly automated driving, value alignment, adversarial robustness

## Abstract

Remarkable progress in the fields of machine learning (ML) and artificial intelligence (AI) has led to an increased number of applications of (data-driven) AI systems for the partial or complete control of safety-critical systems. Recently, ML solutions have been particularly popular. Such approaches are often met with concerns regarding their correct and safe execution, which is often caused by missing knowledge or intransparency of their exact functionality. The investigation and derivation of methods for the safety assessment of AI systems are thus of great importance. Among others, these issues are addressed in the field of AI Safety. The aim of this work is to provide an overview of this field by means of a systematic literature review with special focus on the area of highly automated driving, as well as to present a selection of approaches and methods for the safety assessment of AI systems. Particularly, validation, verification, and testing are considered in light of this context. In the review process, two distinguished classes of approaches have been identified: On the one hand established methods, either referring to already published standards or well-established concepts from multiple research areas outside ML and AI. On the other hand newly developed approaches, including methods tailored to the scope of ML and AI which gained importance only in recent years.

## 1. Introduction

### Background and motivation

In the field of highly automated driving, non-linear system behavior and an unknown environment can be addressed by AI systems. These systems need to act safely at all times – therefore, AI Safety is an important research need. For many AI systems, especially for systems based on neural networks, classical safety analysis methods can hardly be applied. Moreover, current standards do not address the development of safe AI systems. Consequently, for the safety assessment of AI systems the activities validation, verification, and testing need to be considered. In general, safety is particularly of interest dealing with technologies. The underlying philosophical question: “What risk are we willing to take when we use technology?” has led to the establishment of corresponding targets and process specifications in the safety standards (MIT, 2021). The question of trust in the safety of a system is susceptible if it is controlled—fully or partially—by an AI controller. In recent years, in particular, data-driven AI solutions gained importance for controlling safety-critical systems. This also applies to the area of automated driving, where driver-less vehicles operated by machine learning techniques are already a reality, as shown in Krafcik (2020).

Citing Rudner and Toner (2021), machine learning systems do not come with safety guarantees and there are no means yet to ensure that these systems can be operated with a very small risk of failure. Hence, methods providing safety of AI systems and approaches for their validation and verification are needed. The following questions arise: How can AI applications adequately become safer and resilient against undetected development errors and system failures during operation? Can conventional methods be used to develop safe AI applications—in which use cases do new solutions need to be found? Regarding the latter, there are use cases where conventional algorithms can be used in areas wherein an AI solution can be applied safely. As an example, consider a friction clutch. The pressure allowed to close it and whether it can be closed at all can be determined by conventional algorithms. An AI approach could then be designed to find an optimal filling process in terms of comfort and wear of the gearshift. In many use cases, such a dual approach can hardly be applied. For example, if we think of trajectory planning, it seems difficult to monitor an AI solution with a conventional approach. The number of different ways to plan trajectories is too large, and the problem itself too general. AI solutions and classical solutions are combinable and can be mutual beneficial, but it is challenging to identify suitable applications allowing both approaches to fulfill their potential.

### Contribution

The contribution of this paper is an overview of the current research of AI Safety. In order to identify the most relevant categories and topics of this field, a systematic literature review following (Brereton et al., 2007; Kitchenham and Charters, 2007; Liberati et al., 2009; Schumann et al., 2020) was applied. Based on the results of this review process, two categories are identified and discussed: the so-called classical approaches and the new approaches. The section on classical approaches examines ways of how established norms and standards address the problem class of AI Safety. Under new approaches concerning validation, verification, and testing, we summarize newly developed approaches for the safety assessment tailored to the needs of AI use cases. In addition, models for the robust development of AI systems are taken into account.

### Outline

**Figure 1:**
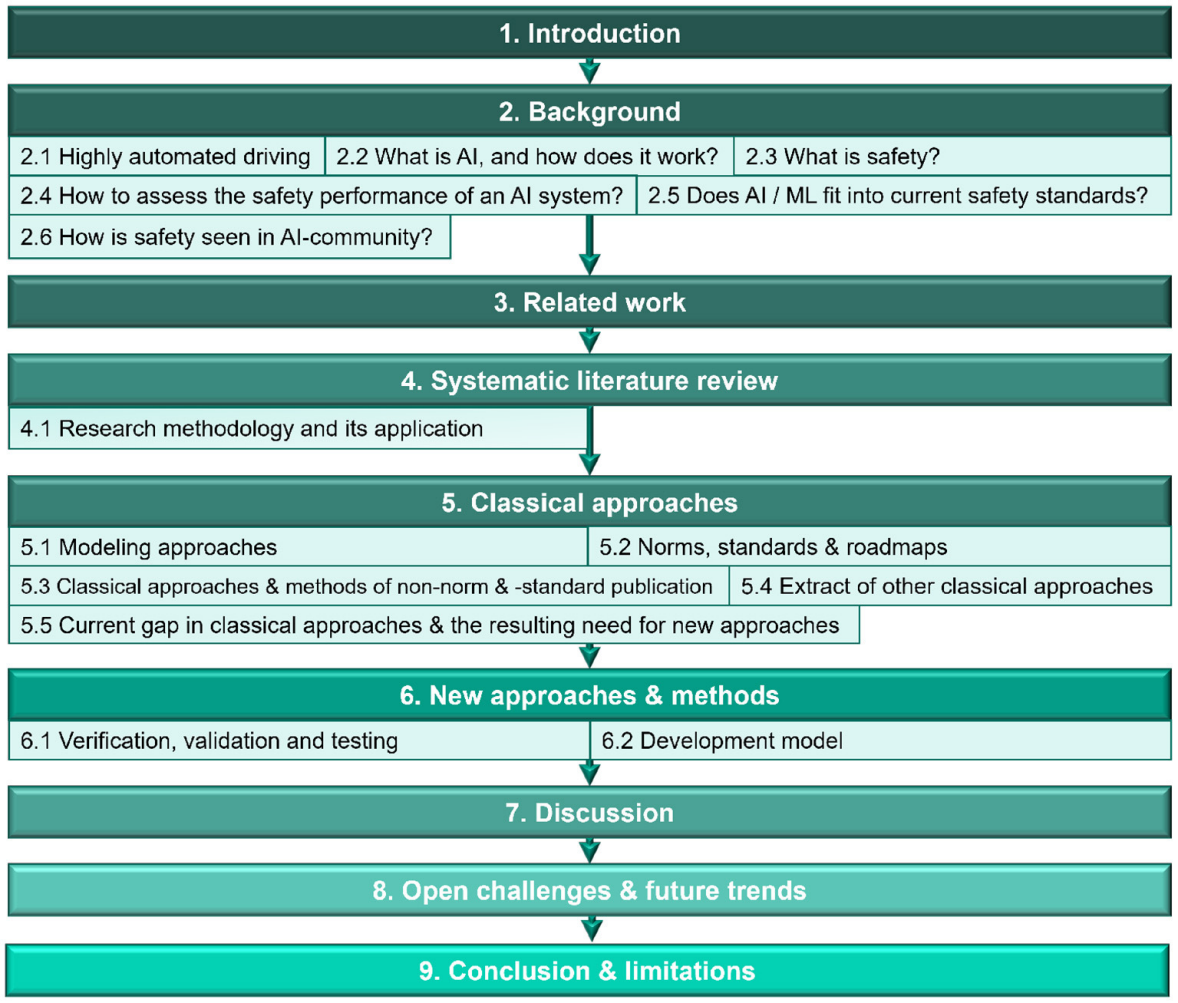
Structure of the review.

This paper is divided into nine sections (see Figure 1). After the “Introduction” in Section 1, Section 2 examines the main research areas “Highly automated driving” and “AI safety” and links to already existing contributions in these fields. Section 3 considers already published literature reviews for the defined problem class of AI safety of highly automated driving. Section 4 describes the planning and conducting of the “systematic literature review,” which forms the basis of this review paper. Sections 5, 6 deal with “classical and new approaches” in the field of AI Safety based on the elaborated research questions. In Sections 7, 8, the findings of the provided literature review are discussed, and topics that, in the opinion of the authors, require further research are mentioned. Finally, Section 9 summarizes the contribution and limitations of this paper.

## 2. Background

### 2.1. Highly automated driving

Over the last years, there have still been many traffic accidents. Alone in the United States of America, almost 40,000 lives were lost in 2020 (National Center for Statistics and Analysis, 2017; Stewart, 2022). The automation of driving tasks can improve traffic safety while improving traffic efficiency, environmental impact, and comfort. However, classical safety approaches do not consider Artificial Intelligence in systems. According to the system theory described by Ropohl (2009), a system acts in a context in order to fulfill its purpose. In Systems Engineering, the purpose and understanding of the context are important tasks (Walden et al., 2015). Then, a system solution is developed that can fulfill the purpose. Models of the complex automotive system support its development. However, the models cannot be complete due to the complex dependencies and the linearization of the behavior. Here, two potential classes of problems emerge that can be more effectively solved by AI. These are dealing with non-linear behavior and an unknown environment.

**Problem 1: Non-linear behavior**: This problem class can describe all complicated and complex problems. Schroeder (2010) describes the possible application of neural networks for non-linear systems. However, systems having non-linear behavior are classical problems. Classical problems consider a known system, e.g., a clutch controlling the maximum contact pressure. In these systems, both the purpose and the context are well-defined and do not change. Furthermore, for these problems, the systems have been continuously optimized over the years.

Due to existing non-linear behavior resulting from the underlying physics or the interaction of different system parts, the question arises of whether AI algorithms can improve control.

**Problem 2: Unknown Environment**: This class deviates from the classical approach. In this problem class, the system's purpose is clearly defined, but the system's environment may change.

One example is an in-car lane departure assistant. The task of this system is to keep the car in the specified lane automatically. In doing so, the system must be able to deal with different weather conditions and road markings. Since here, the testing of the function can only be done partially due to the pervasive environmental conditions, not all failures can be identified. Thus, this problem class describes the operation of an unsafe system in an unsafe environment.

**To conclude, the problem classes of non-linear behavior and unknown environment illustrate the need for AI systems. These systems need to act safely; therefore, *AI Safety* is an important research need**.

### 2.2. What is AI, and how does it work?

According to Nilsson (2009) and Russell and Norvig (2022), Artificial Intelligence (AI) is the subfield of computer science dealing with the task of building intelligent entities, which means to enable these entities to function appropriately and with foresight in their environment.

In recent years, data-driven AI solutions, or machine learning (ML) solutions^1^, have gained particular importance. Machine learning deals with systems that use mathematical and statistical methods to extract regularities and patterns from large amounts of data to solve complex decision or control problems. The process of deriving or as well improving a decision or classification rule is called training. Mostly, training corresponds to solving a mathematical optimization problem for data adaptation by (numerically) minimizing or maximizing a loss function.

According to Sutton and Barto (2018) and Frochte (2019), the field of ML can be subdivided into three broad categories: supervised learning, unsupervised learning, and reinforcement learning^2^. In the supervised learning paradigm, ground truth in terms of labeled data is used for the training of the learning system. The objective is to learn the relationships between the data and its labels in such a way, that the system is able to label accurately unseen data. In the field of autonomous driving, solutions based on this approach are, for example, used for tasks like traffic sign detection, pedestrian detection, or road marking detection (Bachute and Subhedar, 2021). Unlike supervised learning, unsupervised learning does not rely on labeled data. Methods belonging to this field seek for hidden and previously undetected patterns or groupings inside a given data set without prior information on the data. In the automotive sector this type of machine learning can be used for example, as shown in Li et al. (2018), for the clustering of vehicle encounter data. The training principle of reinforcement learning techniques is based on the principle of learning through interaction. Due to repeated interaction with its environment, the learning system discovers which actions generate positive or negative feedback to solve a given task. The system is then encouraged to derive a strategy that generates maximal feedback in terms of a numerical reward function. As suggested by Kober et al. (2013), reinforcement learning approaches are particularly suitable for application in the field of robotics. Furthermore, as shown in Folkers et al. (2019), these methods can be used to derive a controller for a self-driving car.

For the mathematical representation of machine learning systems, so-called artificial neural networks (ANN) are often used. From a purely mathematical viewpoint, any ANN is a parameter-dependent function approximator allowing to approximate a broad class of functions arbitrarily accurate (Hornik, 1991). Especially this feature is fundamental for their broad and frequent use. In terms of their design, structure, and functioning ANNs mimic human brains, which also explains their naming. For a detailed description of the concept, see Hecht-Nielsen (1992) and Abdi (1994).

### 2.3. What is safety?

Colloquially, safety is understood as a state of freedom from risk or danger. In technology, several definitions of the term safety can be found:

1. Following ISO Central Secretary, International Electrotechnical Commission (2014), the term safe describes a state to protect against recognized hazards which likely cause harm.
2. In ISO 61508 (International Electrotechnical Commission, 2010), safety is defined as freedom from unacceptable risks. An acceptable or tolerable risk refers to a risk that is tolerated in a predefined context on the basis of current society values.
3. According to MIL 882E (Department of Defense Systems (DoD), 2012), safety is understood as freedom from states which might cause injury, death, illness, damage to or loss of property or equipment or environment.
4. The standard ISO 26262—see ISO Central Secretary (2018c)—focuses on functional safety in the automotive context. It describes functional safety with the lack of unreasonable risk caused by hazards that are caused by the malfunctions of E/E systems.

According to these definitions, one may say that a system is safe if it can be operated free from all the identified and non-tolerable hazards. This definition also applies to systems comprising AI algorithms, making it necessary to assess their safety. Since many AI systems are built upon ANNs and established safety analysis procedures are hardly applicable in this case—see Section 6 for a more detailed discussion - these methods have to be adapted, or novel approaches have to be found.

### 2.4. How to assess the safety performance of an AI system?

Proving safety for AI systems in a rigorous way is a difficult task and still an open problem—see, for instance, Section 6. This makes verification and validation approaches to ensuring safety requirements all the more important.

#### 2.4.1. Verification

As pointed out in Gausemeier and Moehringer (2002), verification colloquially is the answer to the question: Is the correct product being developed? Technically, according to ISO Central Secretary (2015), verification deals with the task of confirming through the provision of objective evidence that requirements have been satisfied. Thus, following Fisher (2007) and Goodfellow and Papernot (2017), verification aims to give confidence that the product was built adequately and that it will not misbehave under a vast range of circumstances. Usually, the process of verification is realized formally. However, for the verification of ML systems, alternative methods are required (see Section 6).

#### 2.4.2. Validation

Informally, by Gausemeier and Moehringer (2002), validation seeks an answer to the question: Is the right product being developed? In a technical context, validation deals with the issue of confirming by providing objective evidence that the requirements for an intended application have been satisfied. In contrast to verification, systems are not validated formally. Rather, appropriate tests are designed and executed for the process of validation.

#### 2.4.3. Testing

Following Ebel (2015), by testing we understand the determination of properties of a system. In particular, testing provides information about the system, which can be used to check whether the system satisfies defined requirements, objectives, or hypotheses entirely, partially, or not.

### 2.5. Does AI/ML fit into current safety standards?

One might be tempted to view machine learning as just a novel paradigm for designing and implementing software components for cyber-physical systems. So the question arises:

**Can ML-based systems be designed, verified, and certified according to current safety standards?**

Salay et al. (2017) analyzed to which degree ML-based systems could satisfy ISO26262. They conclude that while a large portion of the standard could be satisfied, there exists a set of open issues:

1. ML can create new hazards not due to malfunctioning of the component but due to the complex interaction with humans.
2. Due to its novel development cycle, ML-based systems have distinct faults and failure modes.
3. The capabilities of an ML-based system are inherently tied to the quality of the training data-set. However, this data set is—by definition—incomplete.
4. ML systems having a black box character, e.g., systems based on ANNs, violate the call for hierarchical decomposition.
5. ISO26262 mandates specific techniques for software design, verification, and validation. Some of which are only valid for imperative programming languages.

Consequently, we have to conclude that **ML-based systems cannot fully satisfy current safety standards**. In 2018 ISO initiated a standardization project toward AI: ISO/IEC JTC 1/SC 42 Artificial intelligence. Within this project, working group 3 (WG3) focuses on *trustworthiness*. One aspect of the WG3 is to investigate approaches to realize AI systems' *safety* as well as robustness, reliability, resiliency, accuracy, and privacy. In addition, WG3 has a project on AI risk management, which aims at a standard to address certification processes. A good overview of the current state of “AI standardization” within ISO/IEC JTC 1/SC 42 can be found in Zielke (2020). ISO/IEC AWI TR 5469 (Artificial intelligence—Functional Safety and AI systems) addresses precisely the issue of safety. Also, ISO26262 is currently working on its 3rd edition, in which AI/ML will be addressed. Besides these efforts toward standardization, for several essential topics (especially concerning “safety”), the scientific basis is not sufficiently solid yet, as concluded in Zielke (2020) with three current issues:

- “Formal methods for the verification of neural networks or for the assessment of their robustness (Zielke, 2020)” (c.f. Huang et al., 2020).
- “Architectures and training methods for robust solutions based on deep neural networks (Zielke, 2020)” (c.f. Becker et al., 2020).
- “Methods and tools for generating comprehensible explanations for AI-based decision processes (Zielke, 2020)” (c.f. Goebel et al., 2018).

One key aspect of developing safety-critical systems is the assurance case. The safety argument proves that the system is safe and essential for certification. Schwalbe and Schels (2020) highlight a set of key challenges to overcome in order to assure the safety of ML-based systems: (1) powerful solvers, (2) use of expert knowledge, (3) validation of data and model diversity, (4) model introspection with guarantees. The authors highlight the challenges along the safety life cycle and provide a detailed table listing promising approaches and open challenges.

UL4600 (Underwriters Laboratories, 2020) addresses the safety of autonomous driving systems without human intervention. Therefore, the standard relies on a claim-based approach by using assurance cases. Koopman et al. (2019a) build on the UL4600 standard and derives an approach with goal-based safety cases, and feedback loops in the context of autonomous driving.

### 2.6. How is safety seen in AI-community?

The AI community uses the term *AI Safety*. As summarized in Berlinic (2019), AI Safety may conclude that AI is beneficial or detrimental. AI Safety needs research work to ensure it is beneficial. Yampolskiy and Fox (2012) define the term Safety Engineering for Artificial General Intelligence with the aim of creating safe systems. Amodei et al. (2016) describe AI Safety by mitigating accident risk in machine learning systems. Russell et al. (2015) explain safety by complying with the terms verification, validity^3^, security, and control. Besides safety the terms trustworthiness and confidence need to be considered. According to the mission of the Stanford Center for AI Safety, systems with AI must be safe and trustworthy to facilitate their application in society (Barrett et al., 2022).

In the understanding of this paper, AI Safety deals with the interaction in operating systems to ensure a safe operation (cf. Yampolskiy and Fox, 2012; Amodei et al., 2016. Safety mechanisms help to come to safe operating states. Trustworthiness, verification, validation, security, and control need to be considered in the field of AI Safety (cf. Russell et al., 2015; Barrett et al., 2022).

In the following, seven main challenges in the context of beneficial AI are described (Berlinic, 2019):

- **Fairness**: Machine learning uses decision-making, which might be biased. For instance, the data set is biased due to human prejudices. “AI safety asks: *How do we build AI that is unbiased and does not systematically discriminate against underprivileged groups?” (Berlinic*, *2019**)*.
- **Transparency**: In many cases it is difficult to understand how ML systems make decisions. Especially when the ML system includes a neural network, there is a lack of explainability of the decisions made by the system. “AI safety asks: *How do we build AI that can explain its decisions? How do we build AI that can explain why it made the wrong decision?” (Berlinic*, *2019**)* (c.f. research field Explainable AI).
- **Misuse**: The algorithms can be maliciously used by people. “AI safety asks: *How do we ensure that AI is only used for good causes?” (Berlinic*, *2019**)*.
- **Security**: AI, like every software, is vulnerable to malicious attacks. This might result in unintended actions of the initial design purpose. “AI safety asks: *How do we prevent malicious actors from abusing imperfect AI Systems?” (Berlinic*, *2019**)*.
- **Policy**: AI has an increasing impact on products and society. “AI safety asks: *How do we ensure that AI benefits all, not only a few? How do we handle the disruptions that will be caused by its development?” (Berlinic*, *2019**)*.
- **Ethics**: AI needs to act under certain ethical standards. Human values are one of the broader goals to limit functionalities. “AI safety asks: *How do we decide the values that AI promotes?” (Berlinic*, *2019**)*.
- **Control/Alignment**: AI must be aligned with the values of the designer so that no misinterpretation can happen. “AI safety asks: *How do we align AI with our values so that it does what we intend, not what we ask?” (Berlinic*, *2019**)* (c.f. research field Value Alignment).

As can be seen, AI Safety is a broad topic that exceeds the scope of a single paper. Hence, in this publication, we will focus on the engineering question: How can AI-based systems be designed and executed so that they do not cause accidents? Here, the term accident is defined as unintended and harmful behavior that may emerge from poor AI design (Amodei et al., 2016). According to Amodei et al. (2016) accidents caused by AI-based systems mainly stem from three issues:

- Having the wrong objective function. The wrong function can result from typical mechanisms like negative side effects or reward hacking (i.e., algorithms quickly use bucks to get unintended rewards).
- Having an objective function which is not affordable to evaluate it frequently. For instance, a cleaning robot might not know how to handle each possible tiny object (suck it in or leave it).
- Undesirable behavior throughout the learning process, e.g., from insufficient training data. For instance, the vehicle's environment is changing with new devices and infrastructure to handle.

## 3. Related work

Table 1 gives an overview of the related works considered in this publication.

**Table 1:** Overview of related publications.

**Type**	**Publication**	**Main topic**	**Covered years**
Survey	Juric et al., 2020	Quantitative review with future trends	1985–2019
Report	European Commission, 2020	Implications of legislations concerning AI	Not defined
Blog	Dawson, 2017	Application to critical infrastructures	Not defined
Blog	Krakovna, 2021	AI alignment	Not defined

In Juric et al. (2020), the authors review the topic of “AI Safety” in a quantitative manner. *Via* a dedicated list of keywords, they queried literature databases (SCOPUS, Web of Science, Google Scholar) in order to see how these topics evolved from 1985 to 2019. The single largest growth is in *interpretability*. Strong growth in the number of publications is also in *AI ethics* and *adversarial robustness*, medium growth is in *value alignment* and *safe exploration*, while only slight growth is in *fairness* and *privacy*. Emerging topics seem to be *distribution shift, safe exploration, interruptibility*, and *reward hacking*. However, a substantial number of ideas in the area of AI Safety is not peer-reviewed published. Nonetheless, a set of future research directions can be seen:

- “One of the most important open problems in **explainability** is that there is no agreement on what an explanation is Juric et al. (2020).” The evaluation of the understanding of explanations to humans is not well explored, and there is still no algorithm that provides both high accuracy and explainability.
- Learning a reward function is essential for the future of the field of **value alignment**. In addition, value discovery is another major topic in this research direction. It is the identification of better reward functions with aligned algorithms.
- In the direction of **AI governance**, there is a lack of concrete policy suggestions.
- The direction of **corrigibility** focuses on an online correction of algorithms. The so-called “corrigible reasoning” deals with the design of agents, so they update their reasoning and do not have the benefits of escaping or manipulating something (Soares and Fallenstein, 2017).
- The direction of **safe exploration and distributional shift** handles detecting and adjusting behaviors of agents to prevent mistakes (Amodei et al., 2016).
- **Adversarial robustness** describes the need to minimize the success rates of adversarial attacks.
- **AI Ethics** differ between cultures and evolve over time (Awad et al., 2018; Hagerty and Rubinov, 2019). There is a need to research moral preferences.

Furthermore, the European Parliament, Council, and the European Economic and Social Committee review the safety implications of legislation concerning AI. For instance, the legislation should contain requirements to address the risks of faulty data input at the design phase and processes to ensure quality. In addition, the report states that a risk assessment is necessary not only before entering a product into a market but also before important changes during the product life-cycle (European Commission, 2020).

Besides Juric et al. (2020) and the report of multiple European institutions, we especially found several blog entries dealing with literature reviews on AI Safety (c.f. Dawson, 2017; Krakovna, 2021). Hence, there is a need for further peer-reviewed literature reviews, specifically in areas that are not covered in Juric et al. (2020) and the EU report.

## 4. Systematic literature review

This chapter presents the applied, systematic process of collecting and reviewing the scientific works on the topic. Besides the approach, the data collected during the review and a summary are provided.

### 4.1. Research methodology and its application

In order to conduct a valuable review of the existing literature and cover the addressed topic, a systematic approach is required. The literature review is based on the process described in Brereton et al. (2007), Kitchenham and Charters (2007), Liberati et al. (2009), and Schumann et al. (2020). In the planning phase, the database is created, based on research questions, one example and keywords. The conducting phase consists of a forward search which establishes the reference list. The following backward search investigates this reference list. The documenting phase in chapter 5 and 6 focuses on the identified, relevant publications.

The following Table 2 provides an overview of six steps which are part of the three research phases of planning, conducting and documenting introduced in Brereton et al. (2007).

**Table 2:** Methodology for the systematic literature review (adapted of Brereton et al., 2007; Kitchenham and Charters, 2007; Schumann et al., 2020.

**Chapter**	**Phase**	**Activity**	**Description**
4.1.1	Planning	Select database	Select based on research questions and keywords
4.1.2	Conducting	Forward search and cluster	Search in database with search phrases and cluster
4.1.3	Conducting	Backward search	Search in references of publications
4.1.4	Conducting	Select relevant publications	Select based on criteria
5	Documenting	Document approaches	Document classical approaches
6	Documenting	Document approaches	Document new approaches

**Figure 2:**
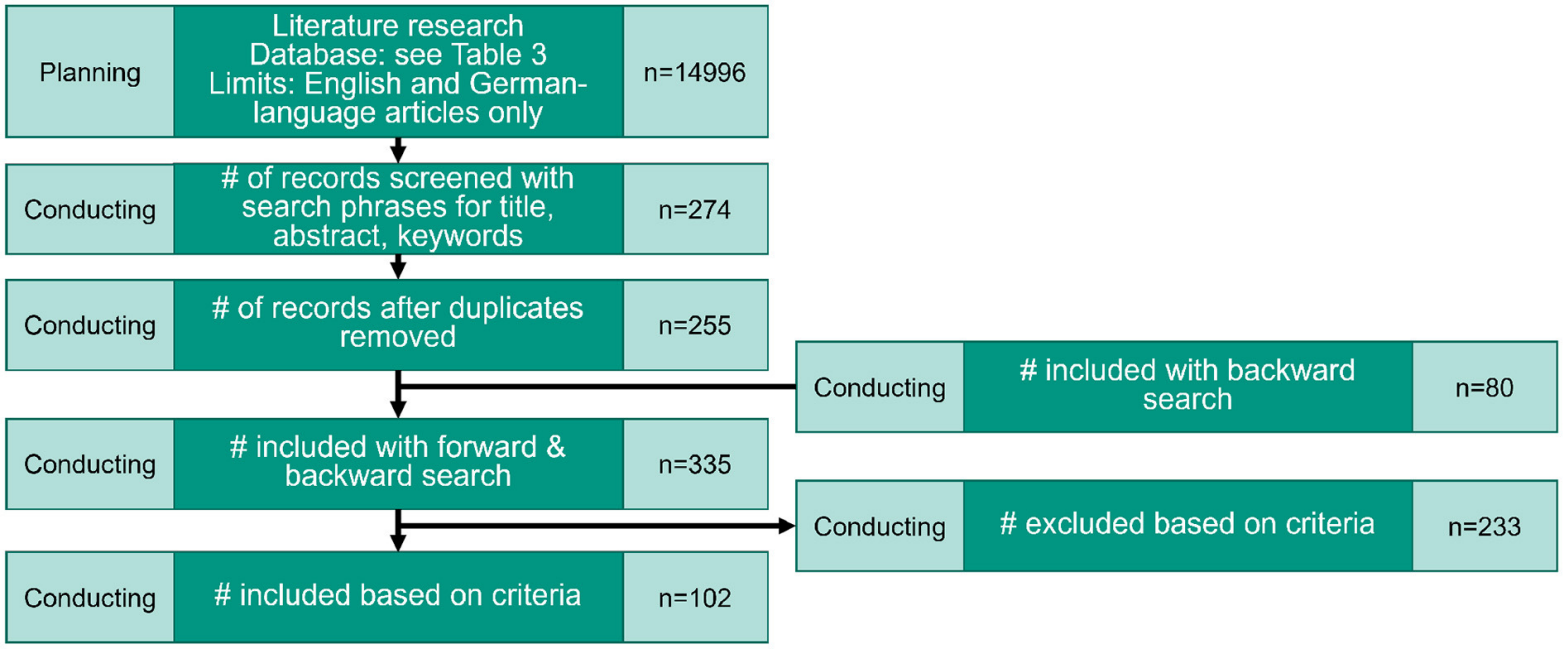
Overview of the number of publications in each activity based on PRISMA (c.f. Liberati et al., 2009).

Figure 2 illustrates the literature search based on the PRISMA (Preferred Reporting Items for Systematic reviews and Meta-Analyses) checklist (c.f. Liberati et al., 2009) with the number of relevant publications in each step of the search process.

#### 4.1.1. Planning phase—Select database

##### 4.1.1.1. Research questions

The methodology starts by formulating the research questions, which are derived from the problem of applying unsafe systems in unknown environments. The answers to the following questions are sought in the context of the example described in the subsection “Example” following “Keywords”:

- What have been the trends in AI over the last 10 years?
- Which are the problem classes for AI Safety in the context of highly automated driving?
- Do suitable validation approaches exist in the identified problem classes?
- Can complex decision-making strategies be learned and tested?

These various questions can be summarized into one guiding question: **Which approaches and methods can be applied for the testing, verification, and validation of AI systems for highly automated driving?**

##### 4.1.1.2. Keywords

**Figure 3:**
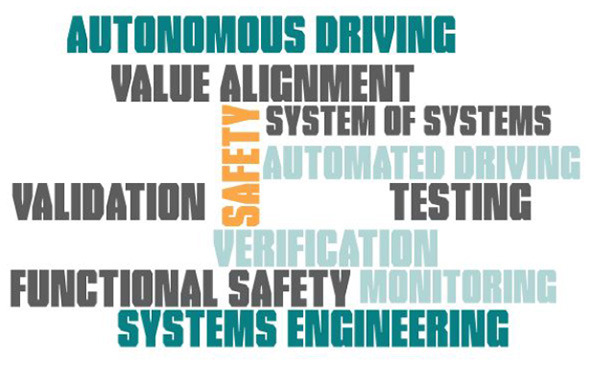
Keywords.

In the beginning, core keywords were identified (see Figure 3). The keywords were substantiated based on the research need and example, as well as on the results in the forward search.

Due to the large number of contributions we have found in this way, we have decided to narrow the focus and exclude the following topics.

- Improvement of systems through AI Algorithms, e.g., sensor fusion.
- AI and Ethics.
- The Security of AI and cyber-physical systems.

##### 4.1.1.3. Example

In order to narrow down the focus of the research, this section describes a reference example.

Another aspect of understanding the challenges *AI Safety* faces is to investigate the intended application in which AI can be used. The question here is whether problems can be derived from the applications that AI can better solve.

In general, the use case is intended to consider autonomous driving. Autonomous driving is the autonomous arrival at a given destination without the intervention of a human being. For this purpose, the vehicle must be able to orient itself in the environment and make independent decisions.

In order to be able to make decisions, the vehicle must be able to identify objects in its environment independently and derive specific actions from them. According to SAE J3016, this corresponds to SAE level 4, i.e., a safety-critical system (SAE International, 2021).

A vehicle is a complex system with multiple subsystems involved in achieving its functionalities from a safety perspective. For instance, the navigation system has the task of suggesting a route to the destination from the vehicle's current position. A control computer is then responsible for controlling the active steering system from the suggested path so that the vehicle reaches its destination. A situation awareness system derives the current situation of the vehicle and the environment from various sensor information. In a hazardous situation, the vehicle must be controlled and stopped by a brake assistant, for example, so that no severe damage is caused. The interaction of various independently acting systems enables the realization of an autonomously driving vehicle.

The vehicle operates in an Operational Design Domain (e.g., public road) and interacts with other systems like traffic participants and infrastructure. This so-called System of Systems can contain AI systems. The typical challenges of System of Systems like different life cycles, operational and managerial independence, and many stakeholders with sometimes conflicting objectives need to be addressed.

In addition, an autonomous vehicle is embedded in other systems. If the position and speed are continuously communicated to a traffic control system, the current traffic situation can be created based on this data. If a forecast is also provided, the navigation system can determine and consider alternative routes. Moreover, newly learned strategies for successfully evaluating and reacting to a situation can be transferred to a fleet of autonomous vehicles. To support the situation representation system, access to external sensors may improve the identification of objects and the system's reaction.

***Thus, an autonomous driving car is a cyber-physical system that interacts with other systems in a highly complex System of Systems***.

##### 4.1.1.4. Database

With import functions on high-ranked journals and identified conferences as well as other publications, a literature basis for the forward search was created. The authors identified that not only journals and conferences but also internet documents contain new and relevant information, which should be considered (see Table 3; c.f. Schumann et al., 2020). The total number of researched publications is 14,996.

**Table 3:** Overview of the database sorted by number of publications.

**Type**	**Publication**	**Timeframe**	**Count**
Journals	IEEE Transactions on Intelligent Transportation Systems	2015–2021	2,400
	Reliability Engineering and System Safety	2015–2021	790
	Journal of Artificial Intelligence Research	2015–2021	360
	Artificial Intelligence for Engineering Design, Analysis and Manufacturings	2015–2021	303
	German Journal of Artificial Intelligence	2019–2021	174
	Frontiers in Artificial Intelligence and Applications	2015–2021	140
Conferences	IEEE Conference on Computer Vision and Pattern Recognition	2015–2021	5,817
	International Conference on Software Engineering	2015–2021	4,742
	International Conference on Artificial Intelligence and Advanced Manufacturing	2019-2021^a^	177
Further Publications	Workshops: WAISE—Workshop on Artificial Intelligence Safety Engineering, AI Safety Workshop	2019–2021	70
	Standards: ISO26262-(1-12:2018), ISO/PAS 21448:2019, ASAM OpenX, ANSI/UL4600-2020	2018–2021	18
	Blog articles^b^	–	5

a Available to the authors on ACM Digital Library.

b Blog articles represent personal opinion without any peer review process. The following blog articles were considered: Ortega et al. (2018), Burton (2021), Gauerhof et al. (2021), Krakovna (2021), and Faculty.

The literature management tool Citavi allows to collect the publications. Afterwards, an analysis is conducted in the tool MAXQDA (VERBI Software, 2021).

#### 4.1.2. Conducting phase—Forward search and cluster

**Figure 4:**
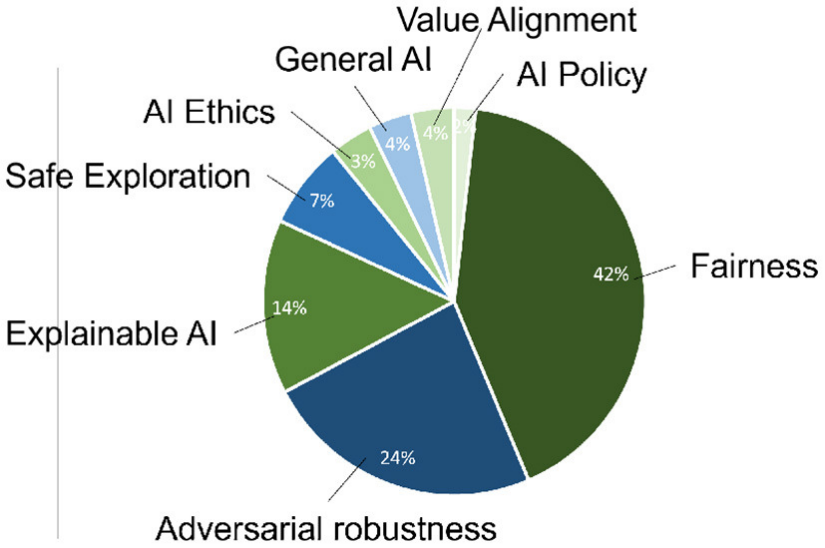
Relative occurrences of categories in database.

In the following, the results of the forward literature search are described with metrics. During the forward search, nine main categories in the field of AI Safety were identified (see Figure 4).

**Figure 5:**
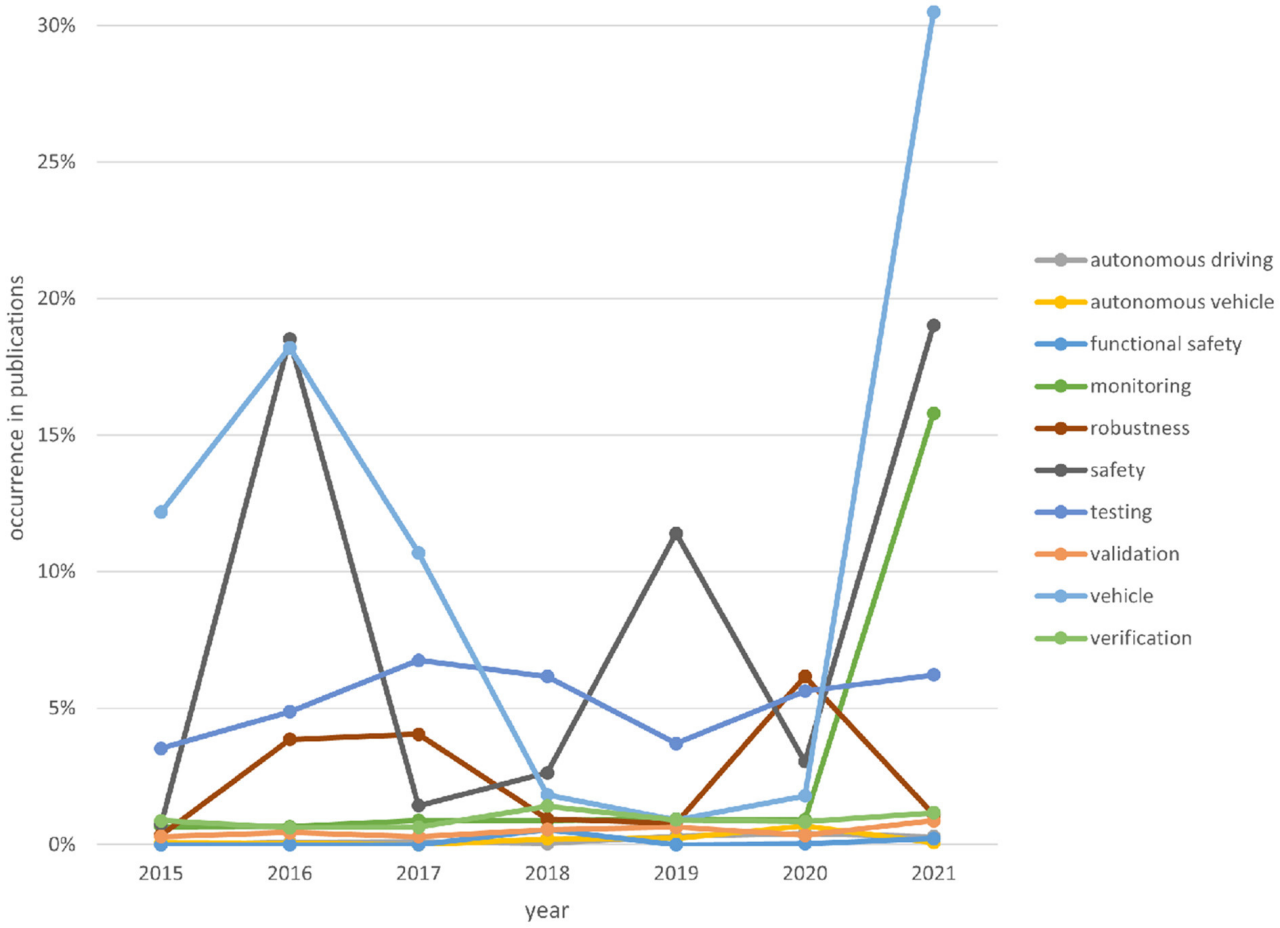
Occurrences of keywords in publications.

Figure 5 shows how often the selected keywords in this contribution are used in the publications over the years. There is high volatility in the usage of certain keywords like robustness. In conclusion, there is no clear tendency. Possible causes are many safety-related documents in 2016 and the tendency to use more specific keywords.

**Figure 6:**
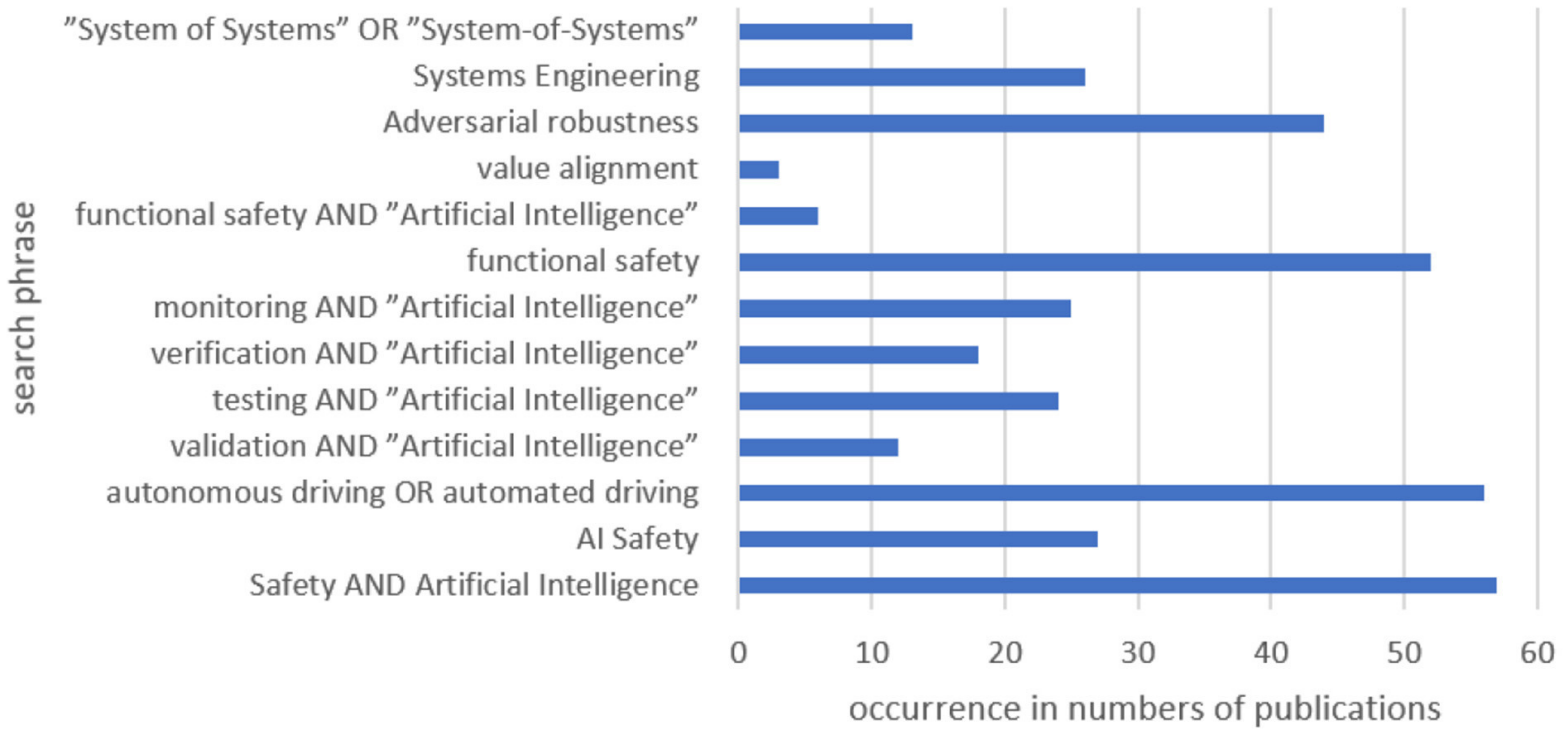
Occurrences of search phrases in publications.

As Figure 6 points out, the literature research resulted in more than 55 relevant documents in the area in focus. As a result, the authors conclude that the amount of publications is sufficient for the forward search phase.

In conclusion, over the past 6 years, there has been a shift of topics more to safety, vehicle(s), and monitoring. Furthermore, the high percentage of certain keywords indicates a good selection of the publications.

#### 4.1.3. Conducting phase—Backward search

The backward search in references of identified publications result in 80 more publications to consider (see Figure 2). Based on the results of the backward search, the list of contributions was divided into the two areas, “established approaches and methods” and “new approaches and methods” (see chapters 5 and 6). The two areas were defined to give a better overview and address the possible use of established approaches and methods.

#### 4.1.4. Conducting phase—Select relevant publications

The final selection of relevant publications are based on the following criteria:

- AI Safety-related
- Selection of high-ranked journals and conferences. Selection of blogs, norms, and standards (see Table 3)
- Identified with search phrases or further references identified with backward search (see Section 4.1.2)
- Suitable for the example of highly automated driving (see example in Section 4.1.1).

Publications related to the criteria “non-English and non-German articles” and the keywords “ethics, security, improvement of system(s)” are excluded to limit the scope.

According to the results of the backward search, the following particularly relevant topics emerged: robustness (6, 27) (read: number of publications in 2015, number of publications in 2021), value alignment (0, 2) and validation (10, 15), verification (16, 30), testing (72, 105).

## 5. Classical approaches

Classical or established approaches describe existing approaches for non-automated and automated systems. For instance, the neural network's relevant approach to neuron coverage resembles the traditional code coverage testing in computer science. Hence, it is categorized as a classical approach. As a result, classic or established approaches are already published standards and established concepts in different research fields over more than 10 years. In the first section, classical approaches are summarized based on model approaches.

### 5.1. Modeling approaches

**Figure 7:**
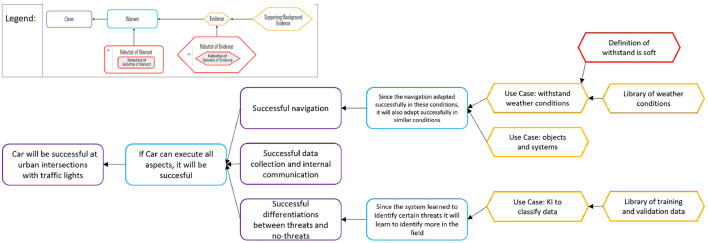
Example classified according to the Toulmin method—extract (c.f. Toulmin, 2003).

Different verification models exist to systematically and formally verify statements of an argument. The field of argumentation theory deals with arguments for logic and rhetoric. An example of a general statement is the claim stated in Figure 7. The already 1958 published Toulmin model with additional content in a new edition (Toulmin, 2003) is shown in Figure 7. This classical approach can handle autonomous learning systems with logic (Collopy et al., 2020). Starting from a claim, one or multiple warrants describe the guarantees for this claim. These can be rebuttals or rely on evidence.

Further extensions are done by Hirata and Nadjm-Tehrani (2019) with a combination of Goal Structuring Notation with the Systems Theoretic Process Analysis to support the safety claim.

In the field of Neural Networks, Kurd's Neural Network Development Model can be applied. It integrates hazard analysis in the development of the neural network's knowledge and deals with neural networks developed specifically for safety-critical use (Kurd and Kelly, 2003).

Shalev et al. introduce a formal model of safe and scalable self-driving cars. The contribution of the paper is two-fold:

Firstly, a mathematical model called “Responsibility Sensitive Safety” (RSS) formalizes an interpretation of “duty and care.” It is designed to achieve the following three goals:

- Its application complies with how humans interpret the law of duty of care.
- The interpretation leads to a useful driving policy, i.e., it should lead to an agile driving policy rather than overly-defensive driving.
- The interpretation is efficiently verifiable so it can be thoroughly proven that a self-driving car correctly applies the interpretation of the law.

Secondly, a semantic language consists of units, measurements, action space, and specifications. The language is used to plan, sense, and actuate an autonomous vehicle. The authors state in particular that the model guarantees (from a planning perspective) that there will be no accidents caused by the autonomous vehicle (Shalev-Shwartz et al., 2018).

Further safety argumentation is realized in Burton et al. (2019) and Schwalbe et al. (2020). Burton et al. (2019) developed an approach for building confidence arguments. These arguments are used for the evaluation of performance in machine learning. The approach was applied for the evaluation of pedestrian recognition.

### 5.2. Norms, standards, and roadmaps

Multiple norms and standards currently address the topic of AI Safety. Furthermore, the domain focus of this contribution is on highly automated driving. For a better overview, the contributions are chronologically introduced. They focus on approaches as well as challenges, guiding questions, requirements, and classifications in the context of AI Safety.

**2018**: For automotive safety, the ISO 26262:2018 addresses functional safety. The norm categorizes the risk by Automotive Safety Integrity Levels (ASIL) to differentiate the proposed measures of handling the risk levels. Inductive methods like the Failure Mode and Effects Analysis (FMEA) have a bottom-up approach to start with the occurred effect first, subsequently deriving the effect's causes. In addition, deductive methods like the Fault Tree Analysis (FTA) use a top-down approach (Salay et al., 2017; ISO Central Secretary, 2018b, p. 11). These result in safety considerations starting from the system and refining it to components with, for instance, their probabilities of default. With the FTA, a probability of default on the system level can be derived.

**2019**: In April 2019, the European High-Level Expert Group on Artificial Intelligence (HLEG-AI) (HLEG, 2019; Independent High-Level Expert Group on Artificial Intelligence, 2020) published Ethical, Legal, and Technical “Key Requirements” for reliable AI systems. The seven requirements include:

- Human Agency and Oversight
- Technical Robustness and Safety
- Privacy and Data Governance
- Transparency
- Diversity, Non-discrimination, and Fairness
- Societal and Environmental Wellbeing
- Accountability.

In a report considering trust in human centered AI, it is stated to realize safety, a fallback plan and a proactive testing of safety measures is necessary. Moreover, safety measures should depend on the risk posed by AI systems. (HLEG, 2019; Independent High-Level Expert Group on Artificial Intelligence, 2020).

In the white paper titled “SAFETY FIRST FOR AUTOMATED DRIVING,” multiple automotive companies address the issue of nonexistent solutions in the topics of automated driving, e.g., safety assurance of AI. They argue that safety-related use cases need to be analyzed for different safety level assurances. Other topics of interest are functional descriptions, assessment, development process, verification, validation, experiments, safety analyses, and safe design (Wood, 2019).

The roadmap SafeTRANS subdivides ten categories for future human-machine systems: integrity and certification, cooperation, context, strength, responsibility, and reflection. For instance, the categories autonomy is subdivided in maneuver, mission, collaborative, and autopoietic autonomy (SafeTRANS, 2019a,b).

Diverse norms rank and categorize future areas of interest according to categories like IT-safety and security (SafeTRANS, 2019a).

**2020**: In 2020, the HLEG-AI presented its final assessment list for Trustworthy Artificial Intelligence. (European Commission, 2020) In total, the group identified four ethical principles and seven requirements which companies can follow to achieve trustworthy AI. For the general safety of AI, the AI HLEG derive five key questions:

- “Did you define the AI system's risks, metrics, and risk levels in each specific use case?”
- “Did you identify the possible threats to the AI system (design faults, technical faults, environmental threats) and the possible consequences?”
- “Did you assess the dependency of a critical AI system's decisions on its stable and reliable behavior?”
- “Did you plan fault tolerance *via*, e.g., a duplicated system or another parallel system (AI-based or ‘conventional’)?”
- “Did you develop a mechanism to evaluate when the AI system has been changed to merit a new review of its technical robustness and safety?”

In contrast to the specific autonomous focus of some norms and standards, the UL4600 is the standard for safety for evaluating autonomous products. The standard gives mandatory and recommended advice in five subcategories for each topic. For machine learning, the standard gives the following six criteria to be considered: acceptable capabilities (1), acceptable performance (2), acceptable data (3), robust to data variations (4), the post-development does not comprise the safety (5), safety also for every other AI not considered in machine learning (Underwriters Laboratories, 2020).

**2021**: Other organizations state questions to address the “future” topic of safety. The European initiatives OSS.5 hosts an event for system safety for SAE level 4 and 5 automated vehicles. On its website, the questions are risen (Tomorrows Business GmbH, 2022):

- How to formulate a continuous safety case in the field of autonomous driving?
- How to integrate safety of functional operations in the field of AI, ML, and Deep Neural Networks?
- How to ensure that deep learning-based systems are safe?

The International Telecommunications Union focuses on “AI for Autonomous and Assisted Driving.” The aim is to develop performance measurement standards for AI that control self-driving vehicles. In particular, a data protocol for Safe AI is in development (International Telecommunication Union, 2022).

### 5.3. Classical approaches and methods of non-norm and non-standard publications

#### 5.3.1. Verification

Cheng et al. (2017) propose extensions for existing safety standards for the usage of neural networks. They extend the aspects “Implementation understandability,” “Implementation correctness,” and “Specification validity” from the existing standard toward safety certification of neural networks. A concrete use case is presented, and a reference to a NASA report covering the topic in the aeronautic area is given (Cheng et al., 2017).

#### 5.3.2. Validation

In the field of validation, Ebert and Weyrich (2019) summarize mainly non-data-driven AI. According to the authors, the validation technologies for autonomous systems can be subdivided into white-box/black-box validation strategy and manual or automatic validation handling. For AI purposes, black-box validation strategies, in particular, should be focused on, as the authors believe AI has a black-box character. As a result of this, the following methods are evaluated in the contribution (Ebert and Weyrich, 2019):

- Experiments and empirical test strategies
- Specific quality requirements tests, for instance, penetration testing, and fuzzing
- Brute-force usage in the real world while running realistic scenarios
- Intelligent validation, for instance, cognitive and AI testing.

#### 5.3.3. Testing

One approach for testing neural networks for autonomous driving with synthetic data is described by the authors (Dreossi et al., 2017). With synthetic data, the authors could test the CNN^4^ to detect cars. This approach can be seen as a classical way of handling the safety analysis by modeling the environment with synthetic data to train the CNN. Combined with further approaches to analyze the robustness of CNNs, this may help generate enough training data. Further research on synthesizing sensor data is done in Yang et al. (2020).

Koopmann introduces safety cases for SAE level 3 testing. He argues that better autonomy results in more challenging situations. The test platform with different actors and systems involved might be partly applicable for higher levels of automated vehicles (Törngren, 2019).

Other research focuses on search algorithms to find (test) data generation in the context of autonomous driving (c.f. Han et al., 2021).

### 5.4. Extract of other classical approaches

An extract of other classical approaches in three different fields is shown in the following.

In the context of **neurological research**, Sotala (2015) researched safe AI with concept learning methods from humans. He reviews multiple approaches and extracts basic steps for concept learning.

The authors' (Page et al., 2018) discuss issues and risks (such as malfunction, malicious attacks, mismatch of objectives) appearing in the usage of AI systems. For this purpose applications of **agent-based systems** are considered. Some of the accompanying risks (and potential strategies for mitigating them) are discussed. These include the change of objectives and the exploration of the environment. This is especially beneficial if the AI has unlimited access to all environment variables. Due to security reasons, this might not be realizable (Page et al., 2018).

In the context of **System of Systems**, the topics of safety and Artificial Intelligence are important. For example, the word “Artificial Intelligence” is used 25 times and safety 121 times in the book “Disciplinary Convergence in Systems Engineering Research.” It is pointed out that a socially acceptable degree of reliability and safety of highly autonomous vehicles can not be assured by treating the vehicle solely as a software system (Koopman and Wagner, 2016). More likely, the vehicles must be seen as part of a System of Systems (Boehm et al., 2018).

### 5.5. Current gap in classical approaches and the resulting need for new approaches

In summary, the classical approaches focus on established approaches in multiple research fields. Classical approaches focus on SAE levels 1–3 and focus on known environments and applications. However, considering higher SAE levels, new approaches are necessary for AI Safety. Moreover, the safety consideration of highly connected AI systems can be further considered. In order to deal with these new challenges of highly autonomous systems like vehicles, the next chapter depicts new approaches for validation, verification, and testing.

## 6. New approaches and methods

Due to the identified gap, new methods are needed. In addition, there is the challenge of autonomous driving (SAE 4) as an application of AI in specification, design, and implementation. In the following, new approaches are described for verification, validation, and testing. Exemplary Adversarial Robustness and Value Alignment are considered in more detail.

### 6.1. Verification, validation, and testing

Many AI systems—for example, ML systems—differ considerably from classical software solutions. Classical software is characterized due to a set of instructions translated into program code by a developer. Applying these instructions to input data gives the output, i.e., it is evident how the output data depends on the input data. In contrast, ML approaches try to extract inference knowledge from so-called training data. If the ML system is based on a neural network, the acquired knowledge is represented in terms of a parameter-dependent model—the neural network.

Neural networks are created by the concatenation of a typically large number of mathematical operations and can schematically be represented in layers consisting of neurons (see, for example, Abdi, 1994). ANNs consisting of multiple layers are called deep neural networks (DNN) (see Montavon et al., 2018). The deeper a network, i.e., the more layers a DNN possesses, the more complex the input and output variables of the network are linked with each other. Consequently, if only the effect of a DNN on given input data is known, i.e., only the corresponding output can be observed, it is difficult to infer the exact functioning of the DNN. For this reason ANNs are often characterized as a black box. According to that, a classical safety analysis is not applicable since a complete understanding of the system's functionality would be required. Additionally, due to the lack of an existing instruction set, traditional validation, and verification methods are not suitable for learning systems. Thus, as requested in Droegemeier et al. (2019), new methods for safety assessment and, in particular, for verification and validation are required.

#### 6.1.1. Verification—adversarial robustness

In recent years, a requirement that has gained importance is robustness against so-called adversarial attacks. It refers to the vulnerability of neural network-based classifiers with respect to small perturbations in the input data. As shown in Moosavi-Dezfooli et al. (2016) or Kong and Liu (2019) at any fixed input sample, for humans, imperceptible perturbations can be constructed such that the resulting perturbed input is misclassified. Inputs of this kind are termed adversarial examples. A taxonomy of adversarial examples and a review of different methods for their generation and as well on countermeasures against adversarial attacks can be found in Yuan et al. (2019). Two trends can be identified for improving adversarial robustness. There are, on the one hand, approaches focusing on the enrichment of the training data-set with a wide variety of adversarial examples (see for example Szegedy et al., 2013; Tramèr et al., 2017; Song et al., 2018) and on the other hand methods based on the adequate choice of the underlying cost function (see Goodfellow et al., 2014; Madry et al., 2017). As addressed in Tsipras et al. (2018), Su et al. (2018) increasing the adversarial robustness of a classifier may negatively affect its accuracy. In contrast to this, however, in Mao et al. (2019) and Stutz et al. (2019), it has been shown that it is, in fact, possible to develop robust and accurate neural network-based classifiers.

Besides developing training strategies to reduce the vulnerability of ML systems against adversarial attacks, research is being conducted to find formal guarantees on its robustness—see for example Hein and Andriushchenko (2017). Formal verification methods, in general, seek to prove that desired properties are satisfied using mathematical reasoning. Even though ANN is a well-defined concatenation of mathematical operations, a formal analysis of its functionality is usually not suitable because of its size and complexity. Hence, citing Katz et al. (2017a), only automatic verification techniques are needed. According to Katz et al. (2017a) again, it can be shown that this problem is nondeterministic polynomial-time complete (or short NP-complete—see for example Goldreich, 2010) and thus difficult to solve. Progress was made in this respect for ANNs based upon ReLU^5^ activation functions—see once more Ehlers (2017), Katz et al. (2017a), or Katz et al. (2017b), Singla and Feizi (2019).

#### 6.1.2. Validation—value alignment

For the validation of ML systems, the area of value alignment is of particular interest and importance. It tackles the issue of developing a system following the intentions of its developer (see Soares et al., 2015; Taylor et al., 2016 or as well Hubinger et al., 2019). To illustrate this issue, consider the fictional cleaning robot problem presented in Amodei et al. (2016). In order to keep an office free from messes, a cleaning robot relying on a ML controller trained by means of reinforcement learning (RL) techniques shall be used. Assume that the training is designed so that the robot gets rewarded only if it is not perceiving any messes in the office. Then the most profitable strategy in terms of reward maximization may be obtained if the robot deactivates its perception system, which ensures that it will not find any messes at all. This, of course, gives a solution that does not solve the actual problem of keeping the office clean. This example emphasizes that training a ML system may result in optimal solutions to the underlying optimization problem but, in general, does not consider other aspects. While harmless in the context of such an example, this phenomenon—called reward hacking (see Amodei et al., 2016)—may have serious implications in other circumstances like the control of self-driving cars, which implies the special relevance of this topic in the given scope. To this end, in Taylor et al. (2016) two main issues are pointed out: specification of the right kind of objective function and the design of ML systems which avoid undesirable behavior even if not perfectly aligned with its developer's intentions. The authors designate and review eight research directions that, according to their conclusions, may be beneficial for the development of reliable and safe learning systems.

The danger that the incorrect choice of the cost function can lead to unexpected and undesirable side effects is also addressed in Amodei et al. (2016). Therein specific attention is paid to the role of RL in the context of value alignment. RL is based on regular interactions between the ML system and its environment, suggesting that this technique can be used as a valid strategy to solve the value alignment problem. However, this assumption is challenged by the possible occurrence of reward hacking, as the cleaning robot example emphasized. A promising extension of RL for tackling the value alignment problem is inverse reinforcement learning (IRL)—citing (Hadfield-Menell et al., 2016) IRL is certainly relevant to the value alignment problem. In contrast to RL, where an optimal control is derived solely by the interaction of the ML system with its environment given a fixed, predefined reward function, approaches to IRL are based, in simple terms, on the idea of mimicking control strategies are considered optimal. More precisely, by observing a system that acts according to an optimal control strategy, one aims to derive the underlying reward function, which is then used to train the ML system. For a more detailed elaboration, see Ng et al. (2000), Hadfield-Menell et al. (2016), or as well Finn et al. (2016).

#### 6.1.3. Testing

Freeman (2020) provides a list of themes that may serve as a starting point for test and evaluation methodologies of data-driven AI systems. In particular, a risk-based test approach is recommended, and the usage of metrics is highly encouraged. Concerning the latter already existing concepts may properly extended or adjusted—see Cheng et al. (2018) and again Freeman (2020). Additionally Skias (2006) suggests tackling during the assessment of a learning system, among others, the following topics:

- Has the correct data been learned, or has been learning something else closely related to the data?
- Did the training procedure give a global optimum or only a local one?
- How does the system react to unseen data or edge cases?

As for non-data-driven systems, any test concept of ML systems has to comprise criteria that allow proving a statement by providing sufficient evidence in favor of this statement. As a procedural approach for this purpose, one may consider again the generic Toulmin-method or, for safety-relevant questions, the approach presented in Kurd and Kelly (2003).

### 6.2. Development model

The increasing usage of applications with machine learning components calls, following Serban et al. (2020), for mature engineering techniques which ensure these are built robustly. As pointed out in Arpteg et al. (2018), there are fundamental differences between developing systems comprising ML solutions compared to traditional software systems. Thus, there is, as pointed out also in Ammar et al. (2006), the need to extend classical software development models like the waterfall methodology or the spiral model. In this regard, the authors of Ammar et al. (2006) discuss three different approaches:

- Common ML system development model: Iterative cycle consists of design, training, and testing stages.
- Rodvold's model: Incorporating nested development loops and containing elements from the waterfall and spiral model.
- Kurd's model: Comprising hazard analysis and addressed in particular for the development of ML systems in safety-critical applications.

An insight into the ML workflow of Microsoft workgroups is provided in Amershi et al. (2019). It consists of nine stages, which are pooled into two groups: data-oriented and model-oriented. The first group deals with data acquisition and data cleaning, while the second group is dedicated, among others, to training, evaluation, deployment, and monitoring. According to findings in Lorenzoni et al. (2021), a very recent work giving a systematic literature review on the development of machine learning solutions, this process is the most comprehensive and accepted one in the literature.

A similar process for the development of ML solutions is provided in Hesenius et al. (2019). In their proposal of a development process, the authors define phases and roles that are to be assigned to members of the development team and are interwoven with the flow of the process. They name here domain expert, data scientist, data domain expert, and software engineer. According to this process, in the first phase, data scientists have to decide whether an ML solution is suitable for the problem. The first model is implemented only after exploring the available data (phase 2), and requirements are defined (phase 3). The last two phases of the process deal with the integration and operation of the developed components. Even though the authors propose an agile development process, they emphasize that the method described can also be applied in a more rigorous process.

## 7. Discussion

Many of the topics discussed in this article can be considered apart from AI Safety in light of other research areas. Specifically, this concerns value alignment and adversarial robustness. While the former can be assigned to the area of AI ethics (see Vakkuri et al., 2019; Kazim and Koshiyama, 2021), the latter is according to Carlini et al. (2019) a security-relevant topic. The importance of security in state-of-the-art vehicle development is discussed, for example, in Schwarzl et al. (2021). The authors discuss the impact of security risks on safety and outline safety and security co-analysis and co-design methods in autonomous driving. From this article, it can be deduced that security aspects must also be considered for a fully comprehensive safety analysis.

A major difficulty in validating and verifying data-driven approaches that should not be overlooked is that all statements about the performance of such an approach are of statistical nature. For example, all that can be said about an ML system for traffic sign recognition is that it correctly recognizes a certain percentage of all signs belonging to a test set. Even if the recognition rate on this test set is one hundred percent, it cannot be assumed that the system works appropriately outside of it, i.e., without false recognition. As was pointed out in Section 6.1.1, even minor disturbances in the input data can lead to misclassifications. Consequently, the quantification of the uncertainty or, more precisely, the specification of an upper bound for the uncertainty with which a ML system makes decisions is highly necessary.

The topic of uncertainty quantification (UQ) is not a unknown one in the science community. The increased use of ML solutions in decision-making has brought this area back to focus in recent years. An overview of the topic is provided for example in Begoli et al. (2019), Abdar et al. (2021), Seuß (2021), or Psaros et al. (2022). In regard to UQ, or more general, in regard to management with uncertainty, we would also like to draw attention on the fuzzy logic approach. According to Zadeh (1983), fuzzy logic or fuzzy reasoning provides an alternative approach for the management of uncertainty. It refers to a multi-valued logic allowing to introduce values between two extreme characteristics like 0 and 1 or true and false. According to Hellmann (2001) and Zadeh (2008), this approach applies human-like thinking in the programming of computers and provides the capability to reason and make rational decisions in an environment of imperfect information. In the context of the (automated) control of a vehicle, fuzzy logic allows for example to formulate and model mathematically notions like steer, steer sharp, brake, or brake hard. For more in-depth information on this topic, its implementation and possible applications, especially in the field of automated driving, we refer to Lee (1990), Passino et al. (1998), Peri and Simon (2005), Dey et al. (2016), and Masmoudi et al. (2016).

## 8. Open challenges and future trends

There is an increasing amount of publications in the area of AI Safety. The authors think this trend will intensify with even more publications in the future. There are promising approaches, especially in the investigated areas of validation/verification/testing. Splitting up the safety assessment of AI systems into the separate tasks of validation, verification and testing seems to the authors as a promising point of reference for future development in the field of AI Safety. The topics of validation/verification/testing will need to be addressed from general perspectives as well as for specific problems.

Several standards and norms in the area of AI Safety of highly autonomous vehicles emphasize the high relevance of the topic. However, as mentioned in Salay et al. (2017), standards like ISO26262 would need to extend their notion of “hazard” toward cases of AI interaction with humans. A first step in this direction can be expected from the publication of ISO/IEC DTR 5469734 Artificial intelligence—Functional safety and AI systems, which is still under development. The authors believe that the introduction of standards or the extension of existing standards to AI systems is essential and one of the prerequisites for the safe application of AI systems in control of safety-critical systems.

Systems consisting of AI are getting increasingly complex. This results in big systems with many interrelations and stakeholders involved. Terms like Advanced Systems, Cyber-Physical Systems and Product-Service Systems can contain AI and need to be safely designed. Hence, the scope of AI Safety should not be limited to one kind of system and authors should consider connectives between systems.

Besides the system's autonomy coming from AI within systems, most systems are socio-technical. Therefore, the interaction with humans is an important topic not covered in this publication. Hereby, multiple standards and norms address this topic and give an outlook (c.f. ISO Central Secretary, 2018a; HLEG, 2019; Koopman et al., 2019b).

The automotive infrastructure is critical due to its many challenging demands for safe and reliable systems. While the main focus is on ground transportation, there are influences and connections to other areas such as aerospace by employing drones or the energy sector by connecting vehicles to a (smart) charging grid. Hence, safety standards and regulations have to be aligned. Classical approaches in other fields like aerospace can be taken into account and can have substantial benefits in the automotive system development. In the context of unknown environments, coping with uncertainties is a crucial task. As already mentioned in Section 7 approaches based on fuzzy logic may be helpful in this regard.

## 9. Conclusion and limitations

Due to the rising number of machine learning solutions and more general AI solutions for the partial or full control of safety-critical systems, the field of AI Safety becomes increasingly important. For the purpose of providing an overview of this extensive research area, a systematic literature review focusing on the field of highly automated driving was conducted. It appears that the topic of AI Safety has become more important over the last years. Despite fluctuations, we identified a trend of publications considering our keywords increasing from 368 (2017), 2,778 (2018) to 2,844 in the year 2021. Furthermore, the review shows that the term AI Safety is only found 12 times. Other search phrases like “AI Testing” or “Safety AND Artificial Intelligence” are mentioned more often. Therefore, it seems that the term “AI Safety” is not yet well-established. The cumulative research question of this paper examines the approaches and methods which can be applied for the verification, validation and testing of AI systems for highly automated driving. According to the findings of this literature review, we identified two major branches comprising approaches and methods for the safety assessment of AI systems: classical approaches, such as the Toulmin method (Section 5) and newly developed approaches (Section 6) tailored to ML use cases, such as value alignment. In addition, the literature review revealed that the aerospace industry has already been facing challenging topics like verification and validation of AI systems for a considerable amount of time—see for example Bhattacharyya et al. (2015) or Underwriters Laboratories (2020). This emphasizes that for the development of safe AI systems in the automotive sector, findings from the aerospace sector should be taken into account and may be used as a guidance. Furthermore, we conclude that approaches for the safety assessment of AI systems can be considered in a general framework superior to the specific use case.

### Limitations

The findings of this work have to be seen in the light of some limitations, which are listed and discussed briefly in the following:

- Six high-ranked journals and three conferences are considered. Furthermore, norms, standards, workshops and blog entries are included, since the topic is developing quickly. Apart from these sources, there might be more relevant research, as identified in the backward research.
- Only German and English language publications were considered. Massive investments in AI research in China (see for example Roberts et al., 2021) suggest putting special emphasis on publications from the Asian region and to consider publications in Asian languages, too.
- The article is clustered in classical and new approaches. Other subdivisions might be helpful and may be considered in future research.
- The research focus with one main research question and nine inclusion keywords and three exclusion keywords can be extended in future research.
- We explicitly excluded the topic of security from the literature research process. As indicated in Schwarzl et al. (2021), a security analysis is indispensable for a comprehensive safety analysis. Thus, an enhancement of the search criteria in direction of security may complete the picture.

## Data availability statement

Whilst not publicly available due to the large amount of data, the data that support the findings of this study is available on request from the corresponding author MW, moritz.waeschle@kit.edu

## Author contributions

MW, FT, AB, and FP made substantial contribution to conception and design and acquisition of data. MW, FT, and AB were involved in analysis and interpretation of data and drafted the article. AA contributed during the revision. All authors contributed to the article and approved the submitted version.

## Funding

A part of the work has been performed in the project AI4DI Artificial Intelligence for Digitizing Industry. This project has received funding from the ECSEL Joint Undertaking (JU) under grant agreement No. 826060. The JU receives support from the European Union's Horizon 2020 research and innovation programme and Germany, Austria, Czech Republic, Italy, Latvia, Belgium, Lithuania, France, Greece, Finland, Norway. In Austria the project was also funded by the program IKT der Zukunft of the Austrian Federal Ministry for Climate Action (BMK). The publication was partly written at Virtual Vehicle Research GmbH in Graz and partially funded within the COMET K2 Competence Centers for Excellent Technologies from the Austrian Federal Ministry for Climate Action (BMK), the Austrian Federal Ministry for Digital and Economic Affairs (BMDW), the Province of Styria and the Styrian Business Promotion Agency (SFG). The Austrian Research Promotion Agency (FFG) has been authorized for the programme management. The funder was not involved in the study design, collection, analysis, interpretation of data, the writing of this article or the decision to submit it for publication.

## Conflict of interest

Authors FT and FP were employed by Virtual Vehicle Research GmbH. The remaining authors declare that the research was conducted in the absence of any commercial or financial relationships that could be construed as a potential conflict of interest.

## Publisher's note

All claims expressed in this article are solely those of the authors and do not necessarily represent those of their affiliated organizations, or those of the publisher, the editors and the reviewers. Any product that may be evaluated in this article, or claim that may be made by its manufacturer, is not guaranteed or endorsed by the publisher.

## Footnotes

1 In the course of this work, the terms machine learning solutions and data-driven solutions are used synonymously. Since machine learning is based on the extraction of regularities and patterns from data-sets, every machine learning solution can also be classified as data-driven. Conversely, there are data-driven methods, for example, in the field of statistics, which cannot be associated with machine learning. Consequently, the terms are to be distinguished in general. In the given context, however, in the opinion of the authors, there is no confusion regarding the meaning of the terms, and therefore the synonymous use of these terms is permissible.

2 In the context of this paper we only aim to give a rough overview of the field of ML and its subdivisions. Therefore, we content ourselves with dividing the field into these three categories. For the sake of completeness, we would like to point out that a finer subdivision can be made. In Russell and Norvig (2022), and also in Sarker (2021) next to supervised learning, unsupervised learning, and reinforcement learning, the additional sub-field of semi-supervised learning is mentioned—in Ayodele (2010) an even finer subdivision is introduced. For an insight into these additional sub-areas, we refer to the literature cited.

3 The authors use the term validity, however in this work we will use the term validation for describing that a system fulfills its purpose.

4 CNNs, short for convolutional neural networks, refer to special artificial neural networks, which are used particularly frequently in the field of image processing. For a brief introduction to the topic, see for example O'Shea and Nash (2015) and Albawi et al. (2017).

5 ReLU, short for Rectified Linear Unit refers to the function *x*∈R↦max(0, *x*). It is a popular choice for activation functions of artificial neural networks—see for example Goodfellow et al. (2016) and Sharma et al. (2017).
